# A multifunctional poly(curcumin) nanomedicine for dual-modal targeted delivery, intracellular responsive release, dual-drug treatment and imaging of multidrug resistant cancer cells†
†Electronic supplementary information (ESI) available: The synthesis procedure of Biotin–PEG–PCDA and the experimental results of MTT. See DOI: 10.1039/c5tb02450a



**DOI:** 10.1039/c5tb02450a

**Published:** 2016-04-19

**Authors:** Jining Wang, Feihu Wang, Fangzhou Li, Wenjun Zhang, Yuanyuan Shen, Dejian Zhou, Shengrong Guo

**Affiliations:** a School of Pharmacy , Shanghai Jiao Tong University , Shanghai , 200240 , China . Email: s.guo@leeds.ac.uk ; Email: srguo@sjtu.edu.cn; b School of Chemistry and Asbury Centre for Structural Molecular Biology , University of Leeds , Leeds , LS2 9JT , UK . Email: d.zhou@leeds.ac.uk ; Tel: +44 (0)113 3436230 ; Tel: +44 (0)113 3436449

## Abstract

A multifunctional anti-cancer nanomedicine based on a biotin–poly(ethylene glycol)–poly(curcumin-dithio dipropionic acid) (Biotin–PEG–PCDA) polymeric nanocarrier loaded with paclitaxel (PTX), magnetic nanoparticles (MNPs) and quantum dots (QDs) is developed. It combines advantageous properties of efficient targeted delivery and uptake (*via* biotin and MNP), intracellular responsive release (*via* cleavable PCDA polymer), fluorescence imaging (*via* QD) and combined PTX-curcumin dual-drug treatment, allowing for overcoming drug resistance mechanisms of model multidrug resistant breast cancer cells (MCF-7/ADR). The PTX/MNPs/QDs@Biotin–PEG–PCDA nanoparticles are highly stable under physiological conditions, but are quickly disassembled to release their drug load in the presence of 10 mM glutathione (GSH). The nanoparticles show high uptake by tumour cells from a combined effect of magnet targeting and biotin receptor-mediated internalization. Moreover, curcumin, an intracellularly cleaved product of PCDA, can effectively down regulate the expression of drug efflux transporters such as P-glycoprotein (P-gp) to increase PTX accumulation within target cancer cells, thereby enhancing PTX induced cytotoxicity and therapeutic efficacy against MCF-7/ADR cells. Taken together, this novel tumour-targeting and traceable multifunctional nanomedicine is highly effective against model MDR cancer at the cellular level.

## Abstract 2

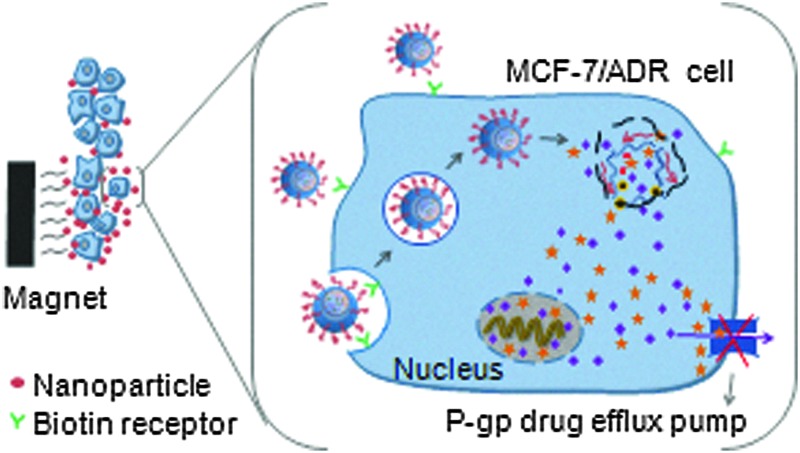
A multifunctional anti-cancer nanomedicine PTX/MNPs/QDs@Biotin–PEG–PCDA was developed aiming at overcoming paclitaxel resistance in MCF-7/ADR breast cancer cells with simultaneous imaging.

## 1. Introduction

Multi-drug resistance (MDR) is a common but one of the greatest challenges currently faced in cancer treatments where cancer cells are resistant to a variety of structurally and mechanistically unrelated chemotherapeutic agents.^1^ MDR tumour cells can effectively remove drugs from their cellular interior to prevent drug accumulation, reducing the sensitivity of tumor cells to therapeutic drugs.^2^ MDR can be caused by both biochemical and physical obstructions such as the over-expression of efflux pumps (P-glycoprotein, P-gp), upregulated pathways (NF-κB and PI3K), reduced penetration of drugs into the cells, and so on.^3,4^

**Scheme 1:**
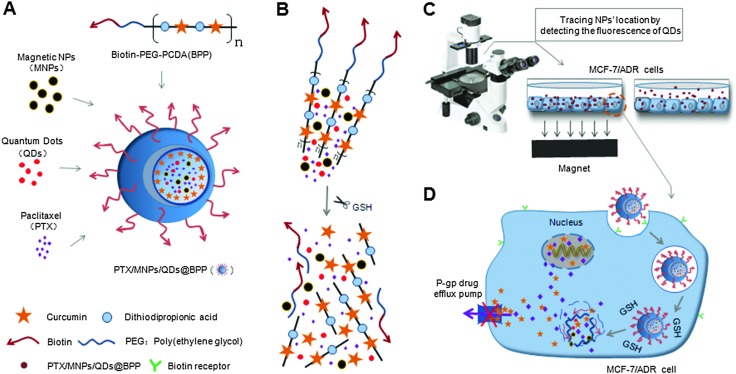
(A) Construction of the PTX/MNPs/QDs@Biotin–PEG–PCDA hybrid multifunctional nanoparticles. (B) The disulphide linkage of the hydrophobic PCDA core can be quickly cleaved by the high GSH content intracellularly, simultaneously releasing its curcumin and PTX payloads. (C) The incorporated magnetic nanoparticles (MNPs) into the multifunctional NP allow for magnetic field guided concentration of the NPs on target cells for increased uptake while the encapsulated QDs allow for sensitive fluorescence detection and tracking. (D) The NP surface biotin groups allow for efficient uptake *via* cancer cell surface over-expressed biotin receptors and subsequently lead to GSH-triggered co-release of curcumin and PTX payloads. Curcumin can down regulate the expression of P-glycoprotein (P-gp) to improve PTX intracellular accumulation and to enhance its cytotoxicity to model MDR cancer (MCF-7/ADR) cells.

An attractive strategy to overcome MDR cancer is to co-administer a specific efflux pump inhibitor along with the cancer chemotherapy drug to increase the drug accumulation and improve treatment efficacy.^5^ Curcumin, a natural diphenol compound extracted from the ground rhizomes of *Curcuma longa*, has shown attractive and selective cytotoxicity towards cancer cells over healthy cells with broad antitumor properties.^6^ More importantly, curcumin is able to independently down-regulate both the PI3K/Akt and NF-κB pathways and suppress the P-gp expression.^7,8^ Therefore, curcumin can be used as an effective sensitizer for MDR cancer cells to increase their therapeutic response to conventional chemotherapeutic agents. However, the extremely low solubility and poor stability of curcumin under physiological conditions have greatly limited its bioavailability and therapeutic efficacy. To overcome this issue, herein we have directly incorporated curcumin into a disulfide-linked hydrophobic backbone of a PEGylated amphiphilic diblock copolymer (biotin poly(ethylene glycol)–poly(curcumin-dithio dipropionic acid)) to improve its stability and water-solubility. Moreover, we have grafted biotin at the hydrophilic end of the copolymer as an active cancer targeting ligand because biotin binds strongly to the biotin receptors which are widely over-expressed on cancer cell surfaces.^9,10^ Importantly, the resulting amphiphilic copolymer (hereafter abbreviated as Biotin–PEG–PCDA, see Scheme 1) can self-assemble into a stable core/shell nanoparticle (NP) in an aqueous environment, acting as an efficient nanocarrier for other cancer chemo-therapeutic drugs (*e.g.* paclitaxel, PTX). As a result, both the chemotherapeutic agent (PTX) and the chemosensitizer can be efficiently delivered into the target cancer cell at the same time.^11^ Importantly, the disulfide linkage at the PCDA hydrophobic section can be readily cleaved by the high intra-cellular glutathione (GSH) content, leading to the co-release of curcumin and PTX intracellularly. The released curcumin can down-regulate the P-gp expression, increasing intracellular PTX accumulation and enhancing its cytotoxicity against the model MDR cancer (MCF-7/ADR) cell lines.^12,13^

Meanwhile, the ability to deliver high drug dose to the target tumour cells can specifically greatly reduce the harmful side effects of most chemotherapeutic reagents.^14–16^ To achieve this ability, we have further loaded Fe_3_O_4_ magnetic particles into the hydrophobic core of the Biotin–PEG–PCDA nanoparticles (NPs), allowing for magnetic field guided delivery of the multifunctional anticancer nanomedicine to target cancer cells with greatly improved efficiency. To further improve the multifunctionality of the nanomedicine, we have encapsulated a non-toxic, Cd^2+^-free CuInS_2_/ZnS core/shell quantum dot (QD) into the hydrophobic core to enable it for traceable delivery. An advantage of using QDs over other fluorescent dyes for traceable delivery is their bright, stable and size-tuneable fluorescence, allowing for sensitive, long-term monitoring and tracking.^17,18^

## 2. Experimental

### 2.1 Materials

A biotin–poly(ethylene glycol)–poly(curcumin-dithiodipropionic acid) copolymer (Biotin–PEG–PCDA, the molecular weights of PCDA and Biotin–PEG–PCDA are 6990 and 10750, respectively) was synthesized as described previously.^19,20^ Hydrophobic CuInS_2_/ZnS quantum dots (QDs) capped with dodecanethiol ligands were purchased from PlasmaChem (German). Paclitaxel was obtained from ZiyunBiotechnology Co., Ltd, (Yunnan, China). 3-(4,5-Dimethylthiazol-2-yl)-2,5-diphenyltetrazoliumbromide (MTT) and dimethyl sulfoxide (DMSO) were purchased from Sigma Co. Ltd (USA). Penicillin–streptomycin, RPMI-1640 medium (R1145), fetal bovine serum (FBS), and 0.25% (w/v) trypsin–0.03% (w/v) EDTA solution were purchased from Gibco BRL (Gaithersburg, MD, USA). All other reagents were at least analytical grade and used without further purification.

Breast cancer cell lines MCF-7 and MCF-7/ADR (multidrug resistance) were kindly donated by the Department of Pharmacology, Chinese Academy of Sciences, Shanghai. The cells were cultured in 75 cm^3^ flasks in a humidified atmosphere with 5% CO_2_ at 37 °C in RPMI-1640 medium supplemented with 10% fetal bovine medium (FBS) and 100 U mL^–1^ penicillin and 100 μg mL^–1^ streptomycin. Cells grown to confluence were sub-cultured every other day after trypsinized with 0.25% trypsin–EDTA and diluted (1/3) in fresh growth medium.^21^

### 2.2 Preparation of magnetic nanoparticles

Fe_3_O_4_ MNPs were prepared by using the co-precipitation method as described previously.^22^ Briefly, FeCl_3_·6H_2_O and FeCl_2_·4H_2_O (2 : 1 molar ratio) were dissolved in de-oxygenated pure water, into which NH_3_·H_2_O was then added dropwise under vigorous stirring and heated to 80 °C to yield Fe_3_O_4_ MNPs. Then oleic acid (∼10% of the mass of MNPs) was added to cap the MNPs rendering them hydrophobic. The resulting Fe_3_O_4_@QA were washed repeatedly by ethanol and isolated by using a permanent magnet. The size and morphology of the MNPs was measured by transmission electron microscopy (TEM) on a JEM-2100F TEM (JEOL, Japan). Their IR spectra were recorded using a Bruker EQUINOX 55 Fourier transformed infrared spectrophotometer (FT-IR, Germany) to detect the surface coating functional groups.

### 2.3 Preparation of PTX-loading PTX/MNPs/QDs@BPP NPs

The PTX-loaded nanoparticles were prepared using the O/W emulsion solvent evaporation method following our previously established procedures. Briefly, a mixture of 10 mg MNPs, 5 mg QDs, 2 mg PTX and 20 mg Biotin–PEG–PCDA was co-dissolved in dichloromethane to form a uniform solution, which was then slowly poured into deionized water under sonication to form an oil-in-water emulsion. After stirring at room temperature for six hours, the organic solvent was rotary evaporated at 40 °C under reduced pressure to obtain a suspension, which was then centrifuged at 3000 rpm for 20 min to remove aggregated particles and un-encapsulated free PTX. The resulting clear supernatant was lyophilized to obtain the multifunctional PTX/MNPs/QDs@BPP nanoparticles.

### 2.4 Evaluation of particle size and zeta potential

The morphology of PTX/MNPs/QDs@BPP NPs was observed using a transmission electron microscope (TEM, JEM-2100F, JEOL, Japan). A drop of the diluted NP solution was placed on a copper grid, stained with 2% phosphotungstic acid and dried before measurement. The average hydrodynamic size and distribution were measured on a Zetasizer Nano ZS (Malvern Instruments Ltd, UK). Zeta potentials of nanoparticles were measured on Zetasizer Nano ZS/ZEN3600 (Malvern Instruments, Herren-berg, Germany) at 25 °C. Each sample was tested in triplicate.

### 2.5 Evaluation of particle rehydration and dilution stability

To investigate the particle rehydration and stability upon dilution which is crucial for efficient drug carriers, the lyophilized powder was redispersed in deionized water by sonication at a concentration of 1 mg mL^–1^. The average hydrodynamic size and the polydispersity index of the nanoparticle were tested repeatedly to check whether agglomeration occurred during this process. The resulting solution was then diluted by pure water to investigate whether the NPs were stable upon dilution.

### 2.6 Investigation of particle magnetic and fluorescent properties

The PTX/MNPs/QDs@BPP NPs were dispersed in an aqueous solution at a concentration of 1 mg mL^–1^, then a permanent magnet was used to determine their magnetic response. Magnetic NPs were able to be pulled to the side wall by an external magnetic field, but were redispersed quickly and uniformly after the magnet was removed. The fluorescence absorption and emission spectra of the NPs were recorded on a HATACHI F-7000 fluorescence spectrophotometer (Japan) to confirm the loading of the QDs.

### 2.7 Determination of drug-loading parameters

1 mg of the PTX/MNPs/QDs@BPP NPs was dissolved in 2 mL of 50% acetonitrile in water being followed by sonication for 10 min to completely break the assembly. The solution was centrifuged at 10 000 rpm for 10 min and the supernatant was filtered with a 0.2 μm syringe filter. The resulting PTX concentration was analysed by high-performance liquid chromatography (HPLC) equipped with a LC 10ADvp pump and a SPD-10Avp UV-vis detector (Waters, USA). The sample solution was injected at least three times at a volume of 20 μL into a Dikma-ODS C18 column (150 mm × 4.60 mm, 5 μm) preceded by a C18 guard column (Dikma, China). The mobile phase was 50% acetonitrile in water with an elution rate of 1.0 mL min^–1^. Paclitaxel detection wavelength was set at 227 nm. The drug concentration of PTX was estimated against a standard calibration curve established under identical conditions. The drug-loading efficiency (DL) and encapsulation efficiency (EE) were calculated by the following equations:
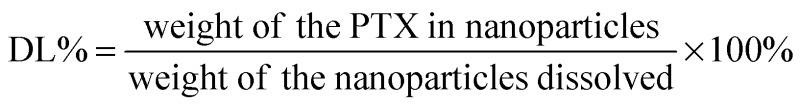
 
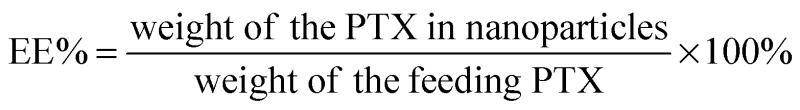


### 2.8 *In vitro* drug release study

The nanoparticle solution was transferred into a dialysis tube (molecular weight cut off = 3500 Da, Snakeskin, Pierce, USA) and suspended in phosphate buffer saline (PBS, 0.15 M NaCl, pH 7.4) containing 1% Tween 80 to imitate a physiological environment. PBS solutions containing 10 μM and 10 mM glutathione were used to mimic the blood and intracellular environment. The release experiments were carried out in an incubator (SHA-C, China) under gentle stirring (100 rpm) at 37 °C. At predetermined time intervals, the release medium was withdrawn and replaced with an equal volume of fresh release medium. The collected samples were analyzed by HPLC as described above to determine the amount of released PTX. Meanwhile PTX release from stock solution was used as control.

### 2.9 *In vitro* fluorescence imaging and cellular uptake study

MCF-7/ADR cells in a logarithmic growth period were seeded at a density of 2 × 10^5^ cells per well in a 6-well plate and incubated overnight. After removing the culture medium, 2 mL of the fresh medium containing the PTX/QDs@PEG–PCDA, PTX/QDs@BPP or PTX/MNPs/QDs@BPP (all containing the same amounts of QDs) were added to each well. The cells were also scheduled to be treated with or without an external magnet. After 4 h incubation, the cells were washed three times with cold PBS to remove unbound NPs, and then imaged on an Olympus IX51 fluorescence microscope (Olympus, Tokyo, Japan) where both fluorescence and bright field photographs were recorded.

For cellular uptake studies, the cells were treated with different NP formulations, PTX/QDs@PEG–PCDA, PTX/QDs@BPP, PTX/MNPs/QDs@BPP, PTX/QDs@BPP plus magnetic field and PTX/MNPs/QDs@BPP plus magnetic field, respectively. At pre-designated time points (2, 4 and 8 h), the cells were washed three times with ice-cold PBS, then collected by centrifugation and then re-suspended in 0.5 mL PBS. The mean fluorescent intensity of the cells was measured on a BD LSRFortessa flow cytometer (Becton Dickinson, America).

### 2.10 *In vitro* cytotoxicity assay

The cytotoxicities of the different NPs were determined by the MTT assay. Briefly, MCF-7/ADR cells in their logarithmic growth were seeded in 96-well plates at a seeding density of 6000 cells per well. Following attachment overnight, the culture medium was carefully replaced with 150 μL of medium containing serial dilutions of the different drug/NP formulations: free-PTX solution (PTX), free PTX + curcumin physical mixture, PTX@PEG–PCDA, PTX@BPP, PTX/MNPs/QDs@BPP and PTX/MNPs/QDs@BPP plus magnetic field. The concentrations of PTX used in the treatment ranged from 0.1 to 100 μg mL^–1^. After incubation for 48 h,15 μL of the MTT solution (5 mg mL^–1^ in PBS) was added to each well. The plates were incubated for an additional 4 h at 37 °C and then the medium was removed. Thereafter, 150 μL of DMSO was added to each well to dissolve the formazan crystals formed. The absorbance of each well was recorded on a Bio-Rad 680 microplate reader (Bio-Rad Laboratories, Hercules, CA) at a set wavelength of 570 nm.

All the cell based experiments were done in triplicate with six parallel samples. Cells treated with culture medium containing 0.1% DMSO were used as control. The cytotoxicity of the drug-loaded NPs was expressed as an IC_50_ value defined as the drug concentration required to inhibit cell growth by 50% relative to the control. These values were calculated by nonlinear regression analysis of the response curves. The cell growth inhibition rate and the reversing drug resistance index on MCF-7/ADR cells were calculated as follows:
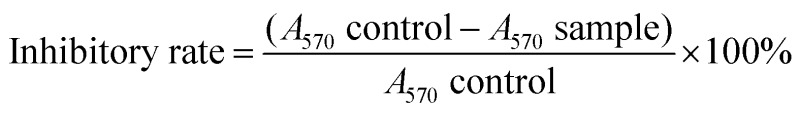
 
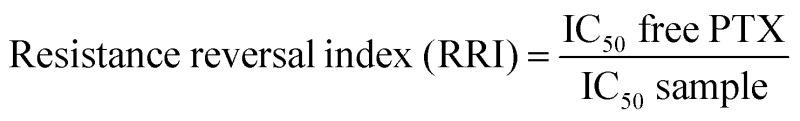


## 3. Results and discussion

Our approach to the multifunctional anticancer nanomedicine is shown schematically in Scheme 1. An amphiphilic biotin modified poly(ethylene glycol) poly(curcumin-dithiodipropionic acid) (Biotin–PEG–PCDA) diblock copolymer was prepared. It was subsequently assembled into stable core–shell NPs in the presence of hydrophobic PTX, Fe_3_O_4_ MNPs and QDs, during which the hydrophobic drugs, MNPs and QDs are encapsulated within the hydrophobic core, forming PTX/MNPs/QDs@Biotin–PEG–PCDA hybrid nanoparticles (abbreviated as PTX/MNPs/QDs@BPP). The encapsulation of the hydrophobic entities significantly increases the nanoparticle stability and structural integrity under physiological conditions *via* enhanced hydrophobic interactions. Moreover, each species brings in a unique function to the multifunctional nanomedicine: QDs for sensitive fluorescence tracing, MNPs for magnetic targeting, PTX for providing chemotherapy, biotin for active cancer cell targeting, PEG for improving water-solubility, stability and resisting non-specific adsorption, PCDA for curcumin incorporation and GSH-triggered intracellular release. Together, all these functional entities allow us to build a novel, multifunctional nanomedicine platform for traceable, targeted delivery, efficient GSH-triggered intracellular drug release and combinational dual-drug therapy for overcoming MDR cancer at the cellular level.

### 3.1 Characterization of PTX/MNPs/QDs@BPP nanoparticles

**Fig. 1:**
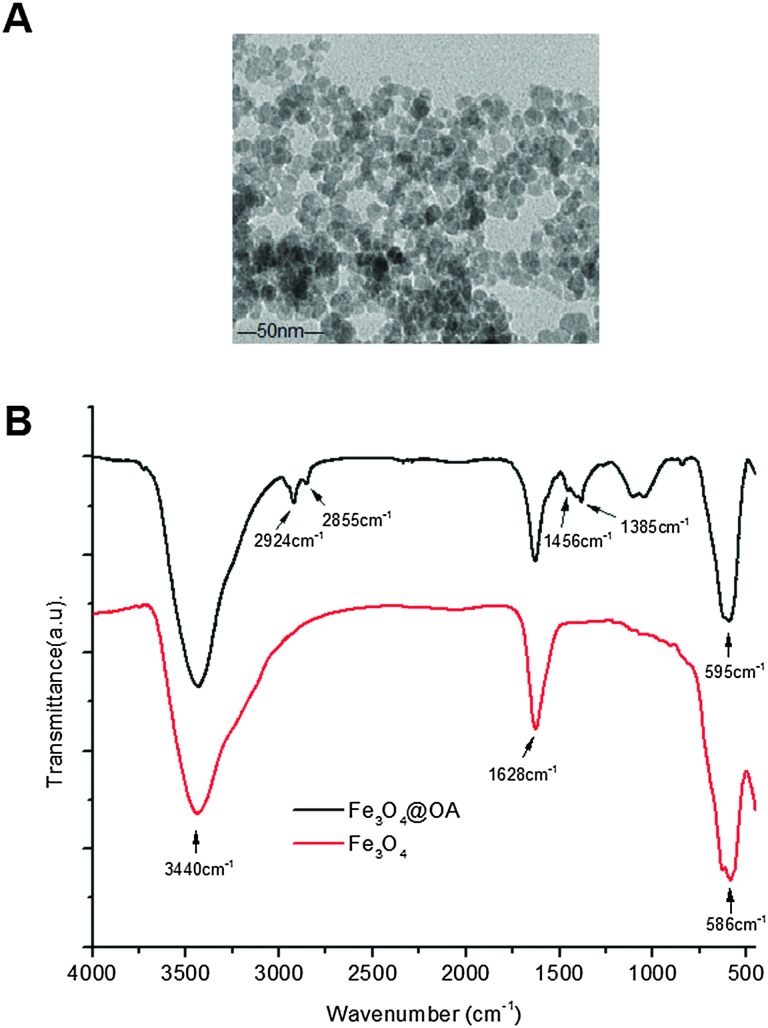
(A) A representative TEM image of Fe_3_O_4_@OA; (B) the FTIR spectra of the bare Fe_3_O_4_ (red) and Fe_3_O_4_@OA (black line).

Fig. 1A shows the TEM image of oleic acid coated Fe_3_O_4_ MNPs used in this study. They mostly appear in a spherical shape with an average MNP core diameter of ∼10 nm. Fig. 1B shows the FT-IR spectrum of the MNPs. During MNP preparation by co-precipitation, the MNP surfaces were readily covered with hydroxyl groups in an aqueous environment. The strong absorption bands at 1628 and 3430 cm^–1^ were assigned to the O–H bending and stretching vibration modes respectively.^23^ Compared with bare Fe_3_O_4_, new absorption bands at 2924 and 2855 cm^–1^ were observed for the oleic acid capped MNPs which were attributed to the existing –CH_2_ asymmetric and symmetric stretching of oleic acid, respectively. The band at 1456 cm^–1^ was attributed to COO^–^ and the band at 586 cm^–1^ was corresponded to Fe–O.^22^ These data indicated that the Fe_3_O_4_ NPs were successfully coated with oleic acid ligands *via* their COO^–^ groups, leaving the alkyl chains exposed to render them hydrophobic. This was essential for loading them into the hydrophobic cavity of the self-assembled amphiphilic Biotin–PEG–PCDA copolymer NPs.

**Fig. 2:**
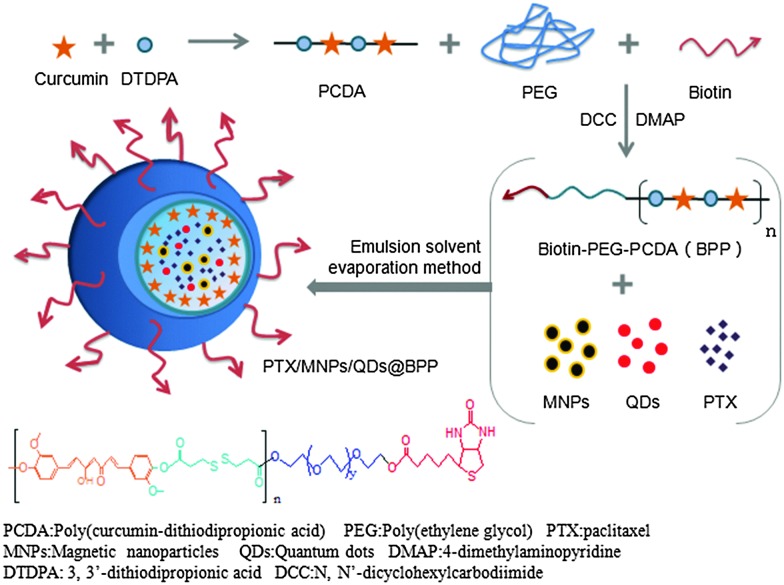
Schematic procedures of synthesising Biotin–PEG–PCDA and the assembly of PTX/MNPs/QDs@BPP.

**Fig. 3:**
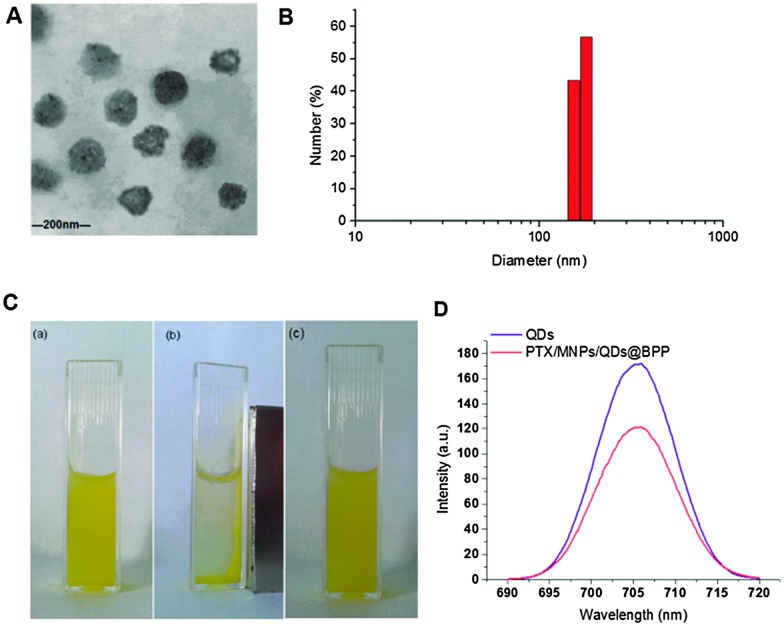
The physicochemical characteristics of PTX/MNPs/QDs@BPP NPs. (A) A typical TEM image of PTX/MNPs/QDs@BPP NPs; (B) histograms showing the size distribution of PTX/MNPs/QDs@BPP NP; (C) photograph of an aqueous solution of PTX/MNPs/QDs@BPP NP showing a uniform yellow colour without any visible aggregation (a), the NPs were rapidly gathered at the side wall of the cuvette by an external magnet (b), the NPs were re-dispersed into a uniform solution after the magnet was removed (c); (D) the fluorescence emission spectra of the free QDs and PTX/MNPs/QDs@BPP.

The preparation procedures of PTX/MNPs/QDs@BPP multi-functional NPs are shown schematically in Fig. 2. Biotin–PEG–PCDA was used to load MNPs, QDs and PTX by the simple O/W emulsion and solvent evaporation method. The success of the multifunctional NP assembly was confirmed by TEM imaging (Fig. 3A): smooth spherical NPs with uniform sizes were clearly observed. The sizes of the NPs were found to be in the range of ∼100 nm, which was smaller than those found by the dynamic light scattering (DLS) shown in Fig. 3B. This is reasonable because TEM image was collected in dehydrated state, where the sizes were mostly corresponded to their inorganic cores and the organic shell was mostly invisible. The zeta potential of the particles was found to be –11.36 mV, consistent with NPs capped with neutral hydrophilic PEGs. Because most of the blood components are negatively charged, the negative zeta potential together with surface PEGs of the NPs should increase their stability by electrostatic repulsion, reducing the chances of particle agglomeration.

The magnetic and optical properties of the NPs were further investigated to examine whether the MNPs and QDs were properly assembled. As shown in Fig. 3C, the NP solution exhibited a yellow colour uniformly, suggesting that they were fully dissolved without aggregation or precipitation. The NPs were rapidly concentrated toward the side wall of the cuvette upon applying an external magnet field, where the solution colour became much less strong, indicating that the MNPs have indeed been successfully encapsulated into the NPs. Therefore, it is possible to guide the NPs by using an external magnetic field. Upon removal of the magnetic fields, the NPs were rapidly re-dispersed into a uniform solution, indicating no agglomeration of the NPs. Fig. 3D showed that the maximum fluorescence emission wavelength of the free QDs and the multifunctional NPs containing the same amount of QDs were almost identical, peaking at 705 and 706 nm, respectively. All these indicated that the QDs were successfully loaded into the NPs, allowing them to be readily detected *via* the strong fluorescence of the encapsulated QDs.^24^

### 3.2 Evaluation of particle rehydration and dilution stability

At first, granules existed when the lyophilized hybrid NP powder was re-dispersed in deionized water. However, the solution became completely clear and uniform after a brief sonication. The average hydrodynamic diameters of the NPs were found to be ∼170 nm with a unimodal size distribution (PDI = 0.25), consistent to those measured by the TEM. The re-dispersed NP powder solution thus showed no significant difference to as-prepared NPs, suggesting that the multifunctional NPs can be conveniently lyophilized and then readily rehydrated and redispersed in water. This indicated that the NPs could be readily lyophilized for convenient long term storage without affecting their structural integrity, a very beneficial property for nanomedicine. To further investigate their stability against dilution, the stock solution (1 mg mL^–1^) was diluted by pure water to give different concentrations, and then the resulting hydrodynamic sizes were measured. As shown in Table 1, the NPs at different concentrations exhibited almost identical particle sizes with a narrow size distribution even at a concentration of as low as 1 μg mL^–1^. Moreover, they also gave very similar negative zeta potentials of about –10 mV. These results indicated that the NPs developed herein possessed excellent stability against dilution and could retain their nanoscale structural integrity even at very low concentrations. Obviously, these results also demonstrated that there was no leaching of the encapsulated quantum dots and magnetic nanoparticles from the nanomedicine, otherwise a range of different sized particles would be observed. Such properties would be extremely beneficial for *in vivo* applications, allowing for the effective minimization of any unwanted premature drug release and degradation and hence reducing side-effects. The outstanding stability of the NPs obtained here is attributed to the strong hydrophobic interactions among the PCDA backbone, hydrophobic PTX molecules, MNPs and QDs at the NP core together with the exposed hydrophilic PEG moieties.

**Table 1:** Particle size and zeta potential of PTX/MNPs/QDs@BPP NPs at different concentrations

Concentration (μg mL^–1^)	Size (nm)	PDI	Zeta-potential value
100	181.1 ± 3.7	0.15 ± 0.04	–12.2 ± 1.8
10	186.5 ± 2.6	0.21 ± 0.06	–11.1 ± 0.9
1	188.7 ± 2.0	0.22 ± 0.01	–9.6 ± 1.4

### 3.3 Paclitaxel-loading parameters

The O/W emulsion solvent evaporation method appeared to be particularly suitable for the incorporation of PTX into self-assembled Biotin–PEG–PCDA NPs. The PTX-loading weight efficiency (DL) and encapsulation efficiency (EE) of PTX/MNPs/QDs@Biotin–PEG–PCDA were determined as 10.3% and 80.7%, respectively. Such high PTX weight loading and encapsulation values were ascribed to the presence of sufficiently large and stable hydrophobic cores of the Biotin–PEG–PCDA copolymer NPs which were further stabilised by hydrophobic interactions with the MNPS and QDs, allowing for the efficient encapsulation of highly hydrophobic paclitaxel molecules.

### 3.4 *In vitro* drug release studies

**Fig. 4:**
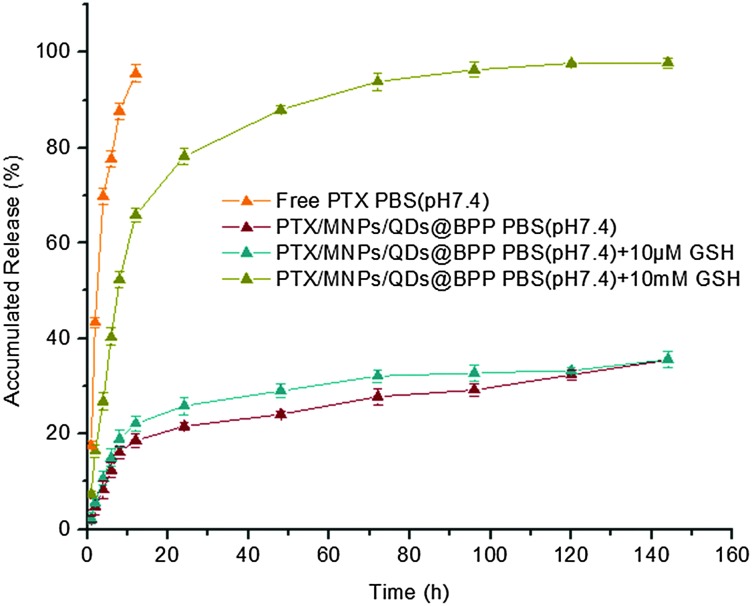
Typical *in vitro* release profiles of paclitaxel from NPs under different simulated conditions at 37 ± 0.5 °C.

In this study, pH 7.4 phosphate buffer solution without and with 10 μM or 10 mM glutathione were selected to imitate physiological, blood and intracellular environments, respectively. The release medium also contained 1% w/v Tween 80 as good sink conditions. In addition, prior to conducting release assays, PTX release from stock solution was investigated as a control. It was found that about 80% of non-encapsulated PTXs were released in approximately 5 h, suggesting that free drugs could freely diffuse through the dialysis membrane. Fig. 4 represents the cumulated *in vitro* release profiles of PTX from the multifunctional NPs in different release medium. In contrast to the rapid release observed from PTX stock solution, a pronounced time prolongation of PTX release from the NPs was evident. For example, only about 25% of the PTX load was released from the NPs in pH 7.4 PBS after 80 h. Importantly, the cumulative PTX release profile showed a strong glutathione dependence: PTX was released much more quickly when exposed to PBS containing 10 mM glutathione, where ∼65% and 90% of drug loads were released at 12 and 48 h, respectively. In contrast, only ∼25% and 30% of drug loads were released with 10 μM glutathione at the same time point. This is mainly due to the effective cleavage of disulfide linkage of the hydrophobic PCDA backbone by GSH, allowing for rapid degradation of the hydrophobic NP core and the release of loaded drugs.^25^ Therefore, the PTX/MNPs/QDs@BPP NPs should be stable under normal physiological or blood circulation conditions (with low GSH content), but could readily release their drug load once entered into the target cancer cells/tissues triggered by the high intracellular GSH content (1–10 mM).

### 3.5 Cell targeting and cellular uptake studies

**Fig. 5:**
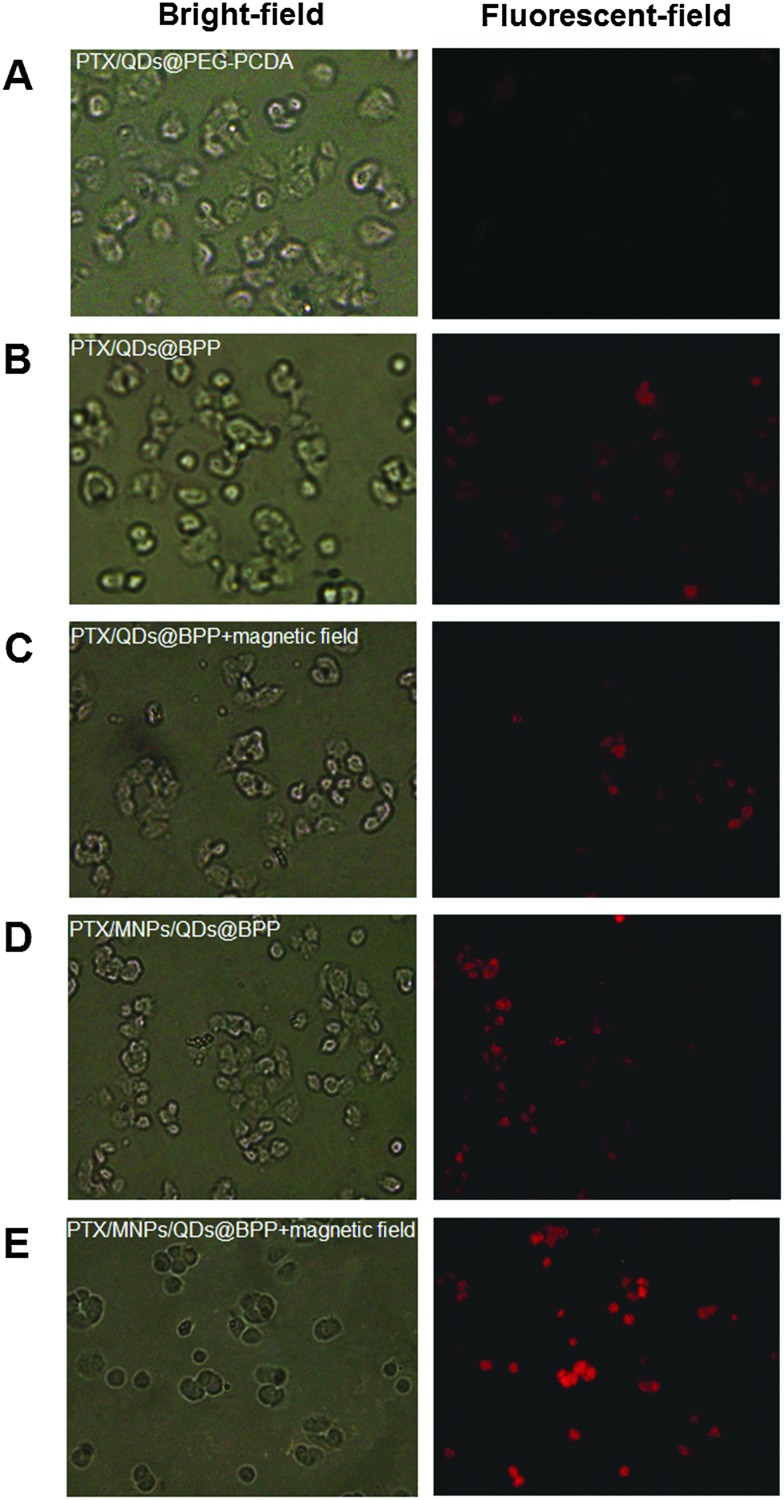
Fluorescent images of MCF-7/ADR cells after incubation with PTX/QDs@PEG–PCDA (A), PTX/QDs@BPP (B), PTX/QDs@BPP plus an external magnetic field (C), PTX/MNPs/QDs@BPP (D), and PTX/QDs@BPP plus an external magnetic field (E).

The encapsulation of strongly fluorescent Cd^2+^-free CuInS_2_/ZnSQDs into the NPs allowed for convenient monitoring of the carrier cell uptake *via* the encoded QD fluorescence by fluorescence microscopy. As shown in Fig. 5A, when the PTX/QDs@PEG–PCDA (without biotin) was incubated with the MCF-7/ADRcells for 4 h, the cells showed minimal QD fluorescence, suggesting minimal cell uptake. The introduction of biotin to the carrier (PTX/QDs@Biotin–PEG–PCDA) was found to improve the cell uptake significantly as evidenced by a strong QD fluorescence (Fig. 5B). This result confirmed that the biotin could act as a targeting ligand to increase cell uptake of the NPs. As shown in Fig. 5C, applying a magnetic field to the PTX/QDs@Biotin–PEG–PCDA (without MNP encapsulation) incubation system appeared to have no effect on the fluorescence intensity of treated cells. However, applying a magnetic field to help pull down the multifunctional NPs from top of the incubation solution was found to significantly increase the fluorescent intensity of the PTX/MNPs/QDs@BPP (containing MNPs) treated cells, where almost every cell was found to display a strong fluorescence (Fig. 5E). The fluorescence intensity was also stronger than that shown in Fig. 5D without applying magnetic field, suggesting that the use of magnetic field for targeted concentration of the NPs is effective in increasing the cell uptake. Taken together, these results revealed that the combination of magnetic and biotin targeting could significantly improve the cell uptake efficiency of the PTX/MNPs/QDs@BPP NPs, demonstrating that the amount of QDs and MNPs encapsulated within the nanomedicine are enough to make them useful for simultaneous fluorescence imaging and magnetic targeting.

**Fig. 6:**
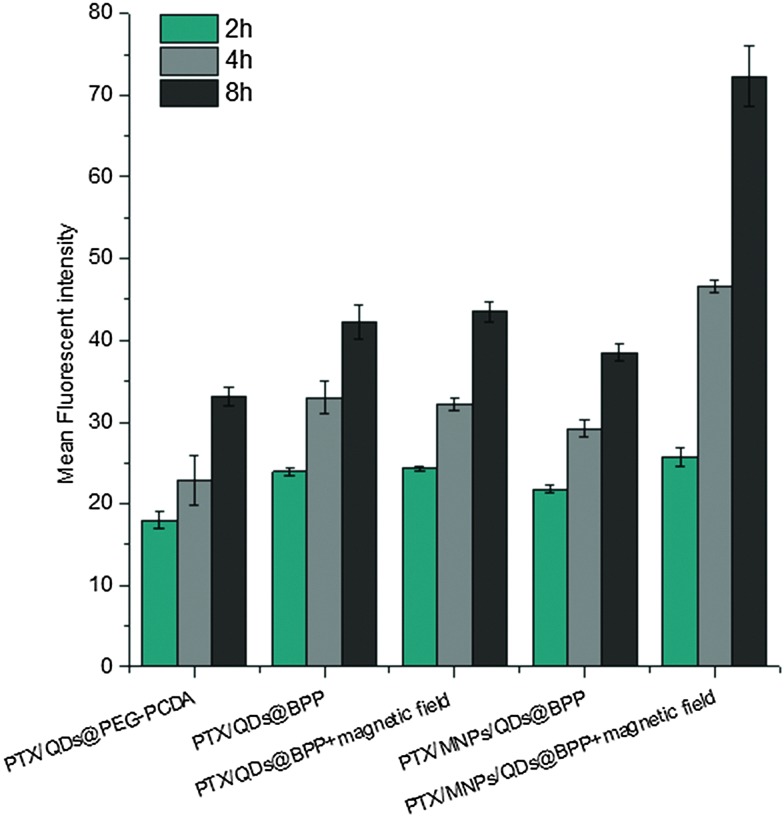
The mean fluorescent intensity of the MCF-7/ADR cells after treatment with different NP formulations with and without applied magnetic field for 2, 4, and 8 h. Data are expressed as the mean ± S.D. (*n* = 3).

To further investigate the magnetic field and biotin medicated cell-targeting effects, the mean fluorescent intensity (MFI) of MCF-7/ADR cells after incubation with different NP formulations for 2, 4, and 8 h was quantified by flow cytometry. As shown in Fig. 6, the MFI of cells was found to increase with the increasing incubation time, consistent with more cell uptake of the NPs with time. Moreover, all of the biotinylated NPs (PTX/QDs@BPP; PTX/MNPs/QDs@BPP) showed higher MFIs than their non-biotinylated NP counterpart (PTX/QDs@PEG–PCDA). This result was consistent with that observed in the fluorescence microscopy imaging experiment described above, confirming that biotin modification on the NP surface can improve cell uptake presumably *via* efficient binding to over-expressed biotin receptors on MCF-7/ADR cell surfaces. Moreover, cells treated with the PTX/QDs@BPP (containing no MNPs) showed no measurable difference in the cellular MFIs in the presence or absence of external magnetic field, confirming that the application of external magnetic field alone did not impact the cell uptake of the NPs. However, a significantly higher MFI (by ∼87% at 8 h) was observed for cells incubated with PTX/MNPs/QDs@BPP (containing MNPs) in the presence of an external magnetic field than that in the absence of external magnetic field, presumably *via* magnetic field guided concentration of the NPs next to the cells. These results indicated that the MNP containing multifunctional NPs were more efficiently taken up by tumour cells from the combined effects of magnet field and biotin dual-modal targeting.

### 3.6 Cytotoxicity assay in MCF-7/MDR cells

**Fig. 7:**
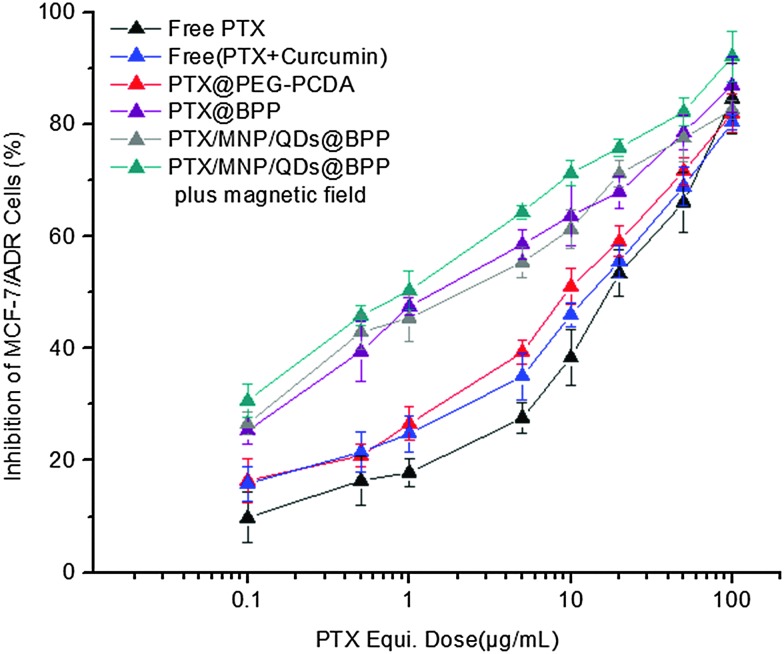
PTX-dose dependent *in vitro* cytotoxicity of the different PTX formulations against the drug resistant MCF-7/ADR cells after 48 h incubation.

Loading of chemotherapeutic drugs into polymeric NPs could increase their cytotoxicity to target cells over free drug.^24,26^ Here the cytotoxicity and drug resistant reversal efficacy of the different formulations of the PTX-loaded multifunctional NPs against the MDR cancer (MCF-7/ADR) cells were evaluated by the standard MTT based cytotoxicity assays. Our previous work showed that the MCF-7/ADR cells were highly resistant to free PTX treatment compared to the PTX-sensitive MCF-7 cell line, displaying a high resistance index of 248. The results shown in Fig. 7 also confirmed the high resistance of MCF-7/ADR cells toward free PTX treatment, giving a high IC_50_ value of 14.9 μg mL^–1^. The combined application of free PTX and curcumin physical mixture reduced the IC_50_ to 9.4 μg mL^–1^. Encapsulation of PTX into the PEG–PCDA NP (*e.g.* PTX@PEG–PCDA) further reduced the IC_50_ to 7.1 μg mL^–1^, suggesting increased cytotoxicity of PTX against the MCF-7/ADR cells. The high resistance of the MCF-7/ADR cells towards free PTX treatment is most likely due to their surface over-expressed P-glycoprotein efflux pumps that can efficiently efflux out of the internalised PTX molecules from the cellular interior, reducing the intracellular PTX concentration and compromising the treatment efficacy. When the MDR cells were treated with PTX-containing PTX@PEG–PCDA NPs, the cleavage of the disulphide linkage of the PCDA backbone triggered by the high intracellular GSH content simultaneously released the curcumin (a degradation product of PCDA) and PTX payloads. Curcumin could then effectively down regulate the expression of the efflux transporters such as P-glycoprotein (P-gp), allowing for increased intracellular PTX accumulation, and thereby enhancing the PTX based chemotherapeutic treatment efficacy.

The combined effect of combinational PTX and curcumin treatment over PTX alone was also evident from the free PTX + curcumin mixture (IC_50_: 9.4 *vs.* 14.9 μg mL^–1^). The different IC_50_ values between the free PTX + curcumin mixture and PTX@PEG–PCDA perhaps originating from the latter can provide the simultaneous intracellular release of PTX and curcumin, maximising the combinational therapy efficacy.^27^ Moreover, the biotin grafted PTX@BPP NP exhibited superior cytotoxicity as compared to the biotin free PTX@PEG–PCDA NPs (IC_50_: 1.68 *vs.* 7.1 μg mL^–1^). This revealed that biotin played a critical role in enhancing cytotoxicity of the NPs, presumably by binding to MCF-7/ADR cell surface over-expressed biotin receptors,^28^ leading to increased cell uptake *via* receptor mediated endocytosis. This result was also consistent with the flow cytometry measurement results, where the biotin-grafted NPs showed higher cell uptake than those without biotin. More importantly, the PTX/MNPs/QDs@BPP multifunctional NPs were the most cytotoxic against the MCF-7/ADR cells in the presence of an external magnetic field among all of the treatment groups. Its IC_50_ value was considerably lower (0.89 *vs.* 1.70 μg mL^–1^) than that in the absence of magnetic field, which again was fully consistent with the much higher cell uptake observed from flow cytometry above. This result clearly demonstrates that the combined magnetic field and biotin dual-targeting strategy was more effective than those relying on biotin targeting alone, a considerable advantage of the multifunctional NPs reported herein over other traditional single-modal targeting NPs.^29^

Table 2 summarised the ability of the above NP formulations to reverse the drug resistance on MCF-7/ADR cells. This was given as the resistance reversion index (RRI), which was defined as the ratio of IC_50_ of free PTX to that of the PTX nanomedicine formulations. As shown in Table 2, treatment with the PTX/MNPs/QDs@BPP in the presence of external magnetic field had a RRI of 16.7 which was significantly higher than any other treatments toward the PTX-resistantMCF-7/ADR cells. This highly encouraging result was benefited by the ability of the multifunctional NP to effectively exploit the magnetic/biotin dual-modal targeting to achieve high cell uptake, efficient intracellular GSH-triggered co-release of curcumin and PTX for simultaneous dual-drug therapy, and thereby maximising the treatment synergy to overcome drug resistance in the MDR tumour cells. Therefore, the PTX/MNPs/QDs@BPP NP developed herein appears to be a highly effective, targeted, and traceable multifunctional nanomedicine for the effective treatment of MDR cancer at the cellular levels.

**Table 2:** IC_50_ values and the drug resistance reversion index (RRI) of various formulations of PTX against MCF-7/ADR cells

Formulations	IC_50_ values (μg mL^–1^)	RRI^*a*^
Free PTX	14.9	—
Free PTX + curcumin	9.4	1.6
PTX@PEG–PCDA	7.1	2.1
PTX@BPP	1.68	8.9
PTX/MNPs/QDs@BPP	1.70	8.7
PTX/MNPs/QDs@BPP		
Plus magnetic field	0.89	16.7

^*a*^Resistance reversion index (RRI): the ratio of IC_50_ for free paclitaxel to that of paclitaxel with reversal agents against the MCF-7/ADR cells.

## 4. Conclusion

A novel magnetic/biotin dual-modal targeting and traceable multifunctional nanomedicine (PTX/MNPs/QDs@BPP) for the efficient treatment of multi-drug resistance breast cancer at the cellular level was developed. It was based on the co-encapsulation of PTX, MNPs and QDs into the hydrophobic core of a self-assembled Biotin–PEG–PCDA block co-polymer. The NPs were highly stable under physiological conditions, but were quickly dis-assembled to release their drug loads in the presence of 10 mM GSH. The NPs were efficiently up-taken by tumour cells from the combined effect of magnet field guided NP concentration and biotin receptor-mediated internalization. It can provide efficient intracellular GSH-triggered release of curcumin and PTX to offer simultaneous dual-drug treatment, leading to significantly improved therapeutic efficacy against multidrug resistant MCF-7/ADR cells. Taken together, the PTX/MNPs/QDs@Biotin–PEG–PCDA NP which combines the ability of fluorescence tracking, MNP/biotin based dual-modal targeting and efficient cell uptake, GSH-triggered intracellular dual-drug release and simultaneous synergistic dual-drug treatment appears to be a novel and effective multifunctional nanomedicine for overcoming MDR in cancer cells. Further work will be focused on exploiting this multifunctional NP for *in vivo* applications.
