# Supplementary material for: A multifunctional poly(curcumin) nanomedicine for dual-modal targeted delivery, intracellular responsive release, dual-drug treatment and imaging of multidrug resistant cancer cells
Source: J Mater Chem B. 2016 Apr 19;4(17):2954–62. doi: 10.1039/c5tb02450a (PMC4847526; doi:10.1039/c5tb02450a)
Supplement: Supplementary file 1 [file TB-004-C5TB02450A-s001.pdf]

Electronic Supplementary Material (ESI) for Journal of Materials Chemistry B.
This journal is © The Royal Society of Chemistry 2016

Electronic Supporting Information

A multifunctional poly(curcumin) nanomedicine for dual-modal targeted delivery, intracellular responsive release, dual-drug treatment and imaging of multidrug resistant cancer cells†

Jining Wang, Feihu Wang, Fangzhou Li, Wenjun Zhang, Yuanyuan Shen, Dejian Zhou, Shengrong Guo

1. **The synthesis procedure of Biotin-PEG-PCDA**

1.1 Materials.

3, 3'-dithiodipropionic acid (DTDPA), N, N'dicyclohexylcarbodiimide (DCC), 4-dimethylamino pyridine (DMAP), glutathione (GSH), methoxyl poly(ethylene glycol) (mPEG, Mw = 5000), poly(ethylene glycol) (PEG, Mw = 6000) and biotin were obtained from Aladdin chemistry Co., Ltd. (Shanghai, China). Curcumin (Cur) was purchased from Alfa-Aesar.

1.2 Synthesis of PCDA.

Cur (1.000 g), DTDPA (0.571 g), DCC (1.150 g) and DMAP (0.100 g) were dissolved in 40 mL anhydrous dichloromethane. This solution was stirred at room temperature for 24 h. the obtained filtrate was added into an excess of anhydrous ether(1000 mL) to produce a precipitate after removing the formed dicyclohexyl urea (DCU) by filtration. The precipitate was further treated multiple times by dichloromethane with anhydrous ether, and finally dried under vacuum to yield a deep yellow solid product (PCDA).

1.3 Synthesis of PEG-PCDA.

PCDA (0.90 g), mPEG (1.125 g), DCC (46 mg) and DMAP (2.74 mg) were dissolved in 40 mL anhydrous dichloromethane. The resulting solution was stirred at room temperature for 24 h. After removing the formed DCU, the filtrate was added into an excess of anhydrous ether, which produced a precipitate. The precipitate was dissolved in water and dialyzed against

deionized water for 24 h with a dialysis membrane (MW cut-off of 6000 Da). The product (PEG-PCDA) was obtained by lyophilization and kept under dry conditions

1.4 Synthesis of Biotin-PEG-PCDA.

Biotin (0.066 g), PEG6000 (1.350 g), DCC (0.111g), DMAP (9.9 mg) were dissolved in 40 mL anhydrous dichloromethane. The resulting solution was stirred at room temperature for 24 h. After removing the formed DCU, the filtrate was added into an excess of anhydrous ether to produce a precipitate, which was further treated multiple times by dichloromethane with anhydrous ether and then dried under vacuum to yield the Biotin-PEG as a white solid. PCDA (0.90 g), Biotin-PEG (0.337 g), DCC (22 mg) and DMAP (2 mg) were dissolved in 40 mL anhydrous dichloromethane. After stirred at room temperature for 24 h and filtration to remove DCU, the filtrate was added into an excess of anhydrous ether to produce a precipitate, which was dissolved in water and dialyzed against deionized water for 24 h with a dialysis membrane(MW cut-off of 6000 Da). After lyophilization, a dry powder ,the Biotin-PEG-PCDA, was obtained.

1.5 Characterization of Biotin-PEG-PCDA

Fig. S1(A) shows the synthetic route to the Biotin-PEG-PCDA polymer. Fig. S1(B) shows the ^{1}H NMR spectrum of Biotin-PEG-PCDA. The product identity and composition were confirmed by comparative analysis of the proton signals.

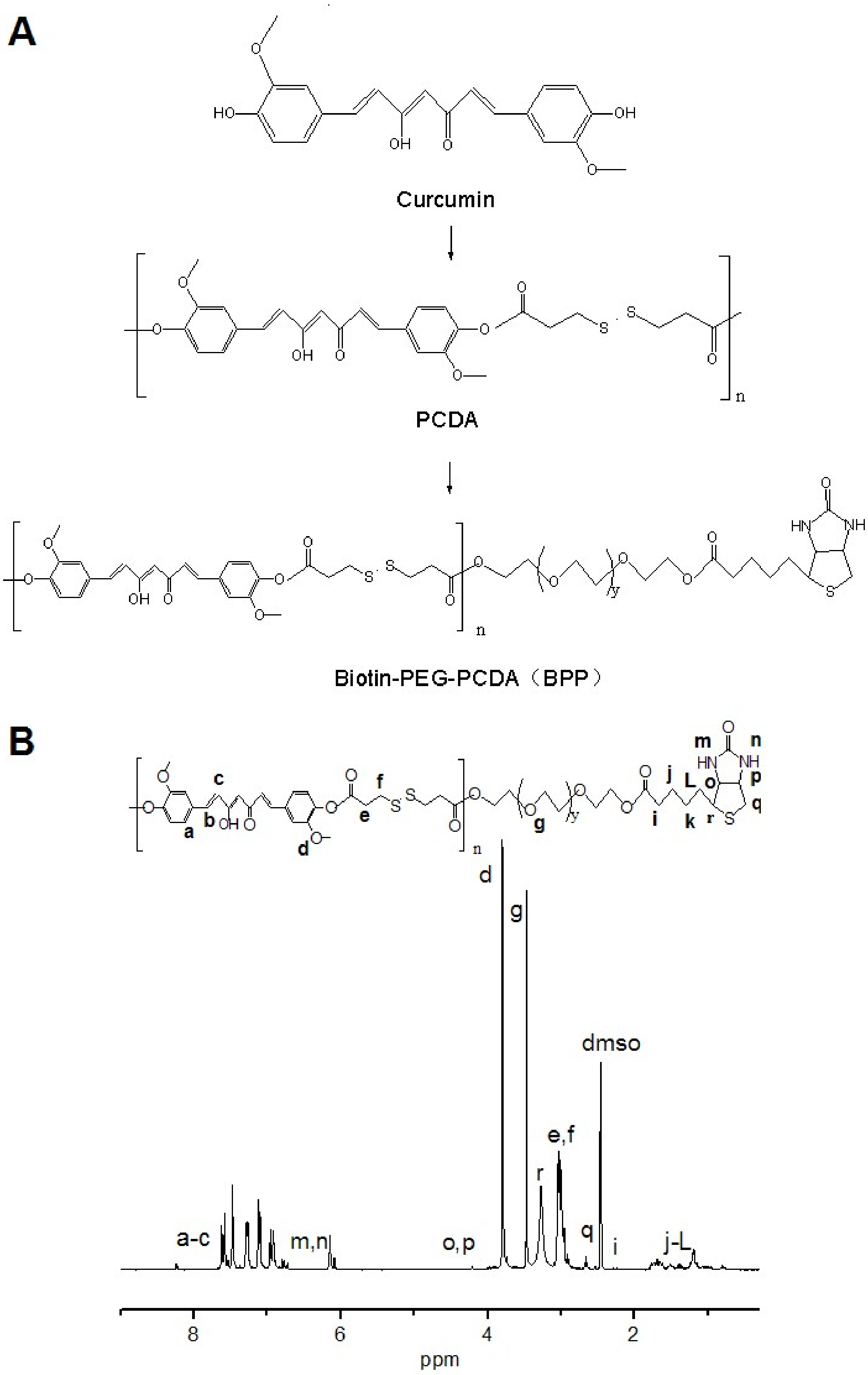

Fig. S1 (A) The synthetic route and the chemical structure of Biotin-PEG-PCDA (BPP); (B) The 1H-NMR spectrum of Biotin-PEG-PCDA.

2. The establishment of paclitaxel concentration standard curve

It was supposed that the concentration of the particle was 20~60μg/ml according to previous experiments, so a Paclitaxel concentration curve (1~200μg/mL) was needed when the mobile phase was a mixture of water and acetonitrile in the volume ratio of 50:50 holding the elution rate at 1.0 ml/min, the volume of injected standard samples were 50 μl constantly and the paclitaxel detection wavelength was set at 227nm using a high-performance liquid chromatography (HPLC). Fig. S2 shows the standard calibration curve for PTX where the X is the concentration of paclitaxel and the Y is the peak area of PTX in the HPLC elution profile.

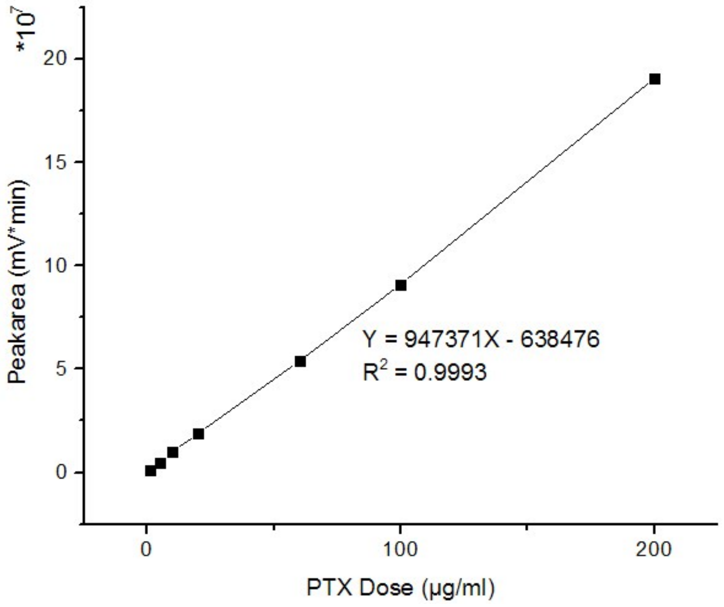

Fig. S2. The standard calibration curve of paclitaxel concentration

3. The research of the multiple MCF-7/ADR cells resisting drug

MTT assay was taken to explore the multiple that MCF-7/ADR cancer cell resists drug in contrast of MCF-7 cancer cell. MTT classical method was taken to explore the level that MCF-7/ADR cancer cell resists drug. Human breast cancer cell line MCF-7 and MCF-7/ADR cells in logarithmic growth were washed and digested to be dispersed by culture medium in 96-well plates at a density of 6000 cells/well respectively. Serials of certain concentrate paxlitaxel were added after incubation. The treatments was replaced with MTT assay after 48h.A microplate reader(Bio-Rad 680,USA)was taken to measure the absorbance of each well, when the test wavelength is 570nm.It is necessary to calculate IC_{50} values which represents the drug concentration required to inhibit tumor cells growth by 50% relative to controls to explore each group of drugs reversing multidrug resistance effects on MCF-7 / ADR cells by following formulas.

$$Inhibitory\ rate(IC) = \frac{(A570control - A570PTX)}{A570control} \times 100\%$$

$$Resistance\ index(RI) = \frac{IC50(MCF\text{-}7)}{IC50(MCF\text{-}7/ADR)}$$

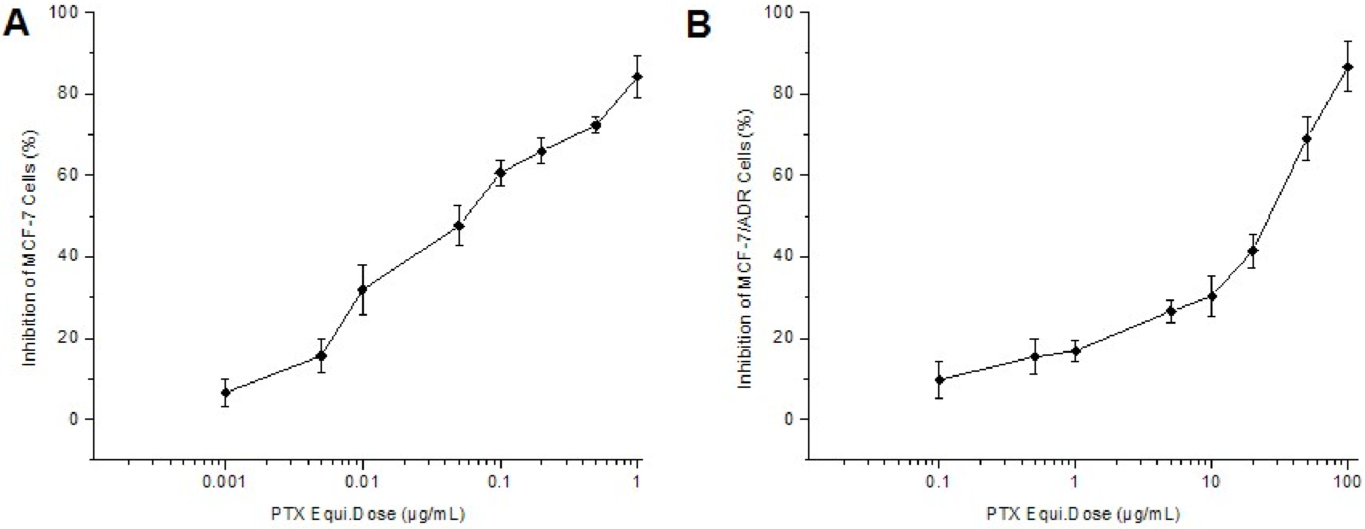

Fig. S3. The inhibition rates of paclitaxel in certain concentrations against (**A**) MCF-7 cells; (**B**) MCF-7/ADR cells after 48h incubation

According to Fig. S3, the half inhibitory concentration (IC_{50}) values of paclitaxel against MCF-7 and MCF-7/ADR cells were determined as 0.06 and 14.86 μg/ml respectively. The resistance index of MCF-7/ADR was 248 following the calculate outcome that IC_{50} (MCF-7) divided by IC_{50} (MCF-7/ADR).

4. The cytotoxicity of the materials, MNPs, QDs and Biotin-PEG-PCDA

The MTT assay was taken to measure the cytotoxicity against MCF-7/ADR cells of the materials, MNPs, QDs and Biotin-PEG-PCDA. Such experiments were taken to explore whether the materials were obviously cytotoxic without the effect of drug. MCF-7/ADR cells in logarithmic growth were seeded in 96-well plates at a density of 6000 cells/well and included for 12 h. Then a series of certain concentration of the MNPs, QDs, and Biotin-PEG-PCDA were added and incubated for 48 h. Cytotoxicities of these different treatments were examined by MTT assay following our established procedures (ref. 25).

Fig. S4 shows the cytotoxicity of materials against MCF-7/ADR tumor cells in the absence of drug. All of them showed slight cytotoxicity under such experiment concentrations. Here the MCF-7/ADR cells were incubated with materials whose concentrations far exceeded those in

the PTX/MNPs/QDs@BPP, the resulting cell viabilities were still stood at 81%, 90% and 80 % after treatment with the MNP, QD and Biotin-PEG-PCDA, respectively. Thus these materials should pose no significant cytotoxicity toward MCF-7/ADR cells under our treatment conditions, which is also fully consistent with other literature results. This experiment verified that it would be safe to ignore the very slight cytotoxicity of the materials, where the inhibition effects on tumor cells were mainly come from the drugs, PTX and curcumin.

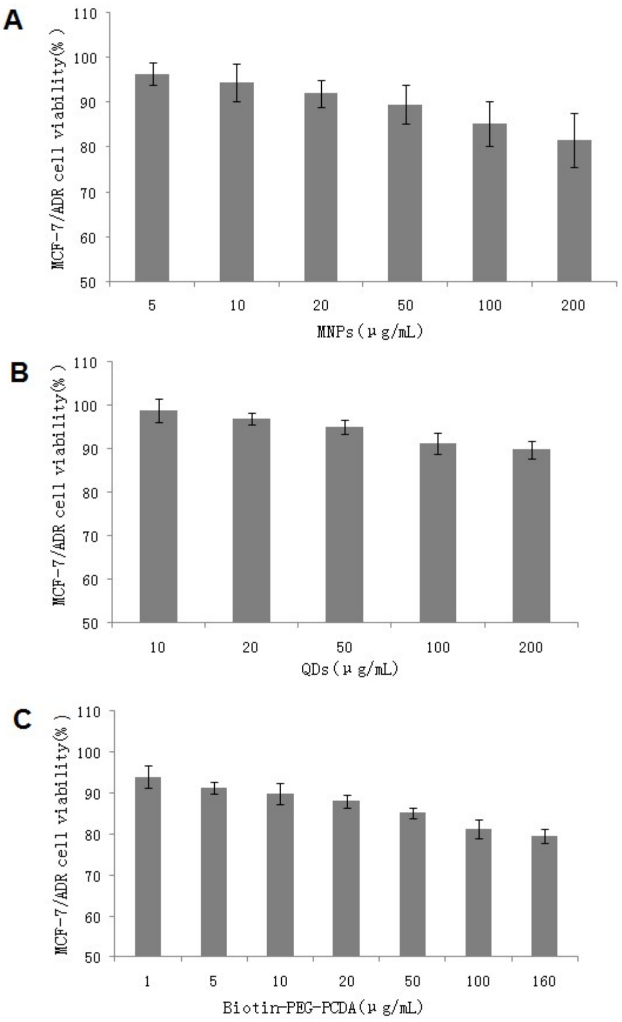

Fig. S4. Cell viabilities of the MCF-7/ADR after incubation with different amounts of the (**A**) MNPs, (**B**) QD and (**C**) Biotin-PEG-PCDA.

Comment [DZ]: It would be useful to indicate what was the maximum concentrations of each component used in multifunctional nanomedicine treatment!

Table S1. Cytotoxicities of PTX, CUR, MNPs, QDs and Biotin-PEG-PCDA against MCF-7/ADR cells

	Concentration (μg/mL)	Cell viability (%)
PTX	14.9	50
CUR	51.8	50
MNPs	100.0	85
	200.0	81
QDs	100.0	91
	200.0	90
Biotin-PEG-PCDA	100.0	82
	160.0	80
